# Supplementary material for: A Bayesian machine learning approach for drug target identification using diverse data types
Source: Nat Commun. 2019 Nov 19;10:5221. doi: 10.1038/s41467-019-12928-6 (PMC6863850; doi:10.1038/s41467-019-12928-6)
Supplement: Supplementary file 1 — Supplementary Information [file 41467_2019_12928_MOESM1_ESM.pdf]

A BAYESIAN MACHINE LEARNING APPROACH FOR DRUG TARGET IDENTIFICATION USING DIVERSE DATA TYPES

Madhukar et al.

SUPPLEMENTARY FIGURES

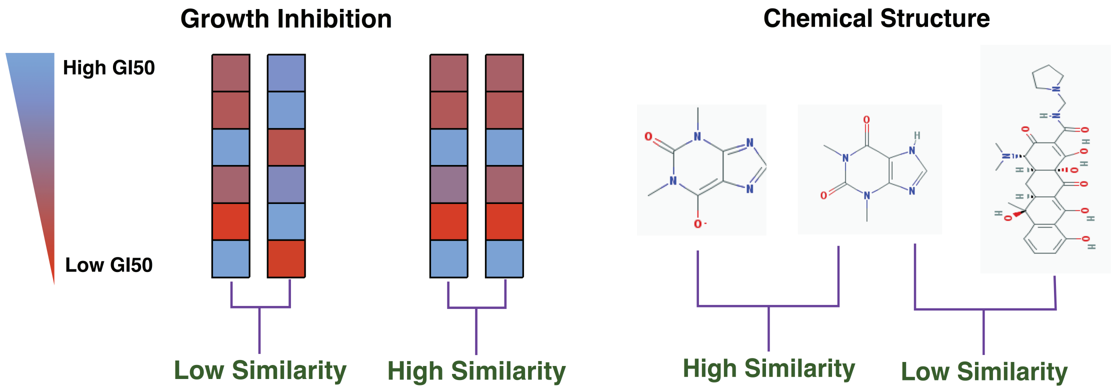

Supplementary Figure 1: Examples of how BANDIT calculates a growth inhibition similarity score and a chemical structure similarity score

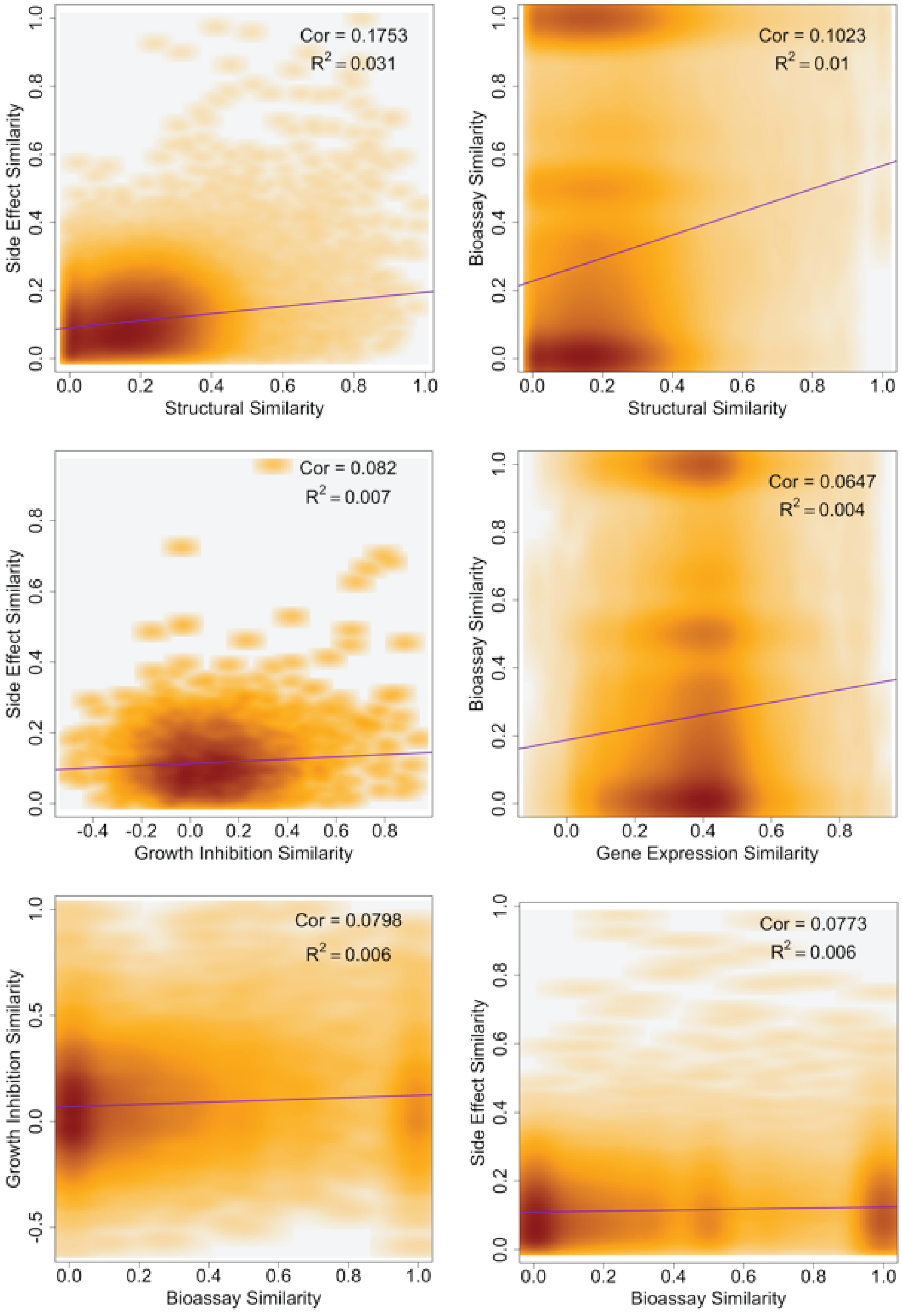

Supplementary Figure 2: Various similarity scores appear to be uncorrelated and independent

Density plots showing how various different similarity scores correlate with one another, with darker area corresponding to a higher density of values. R^2 and P value were calculated using a pearson correlation.

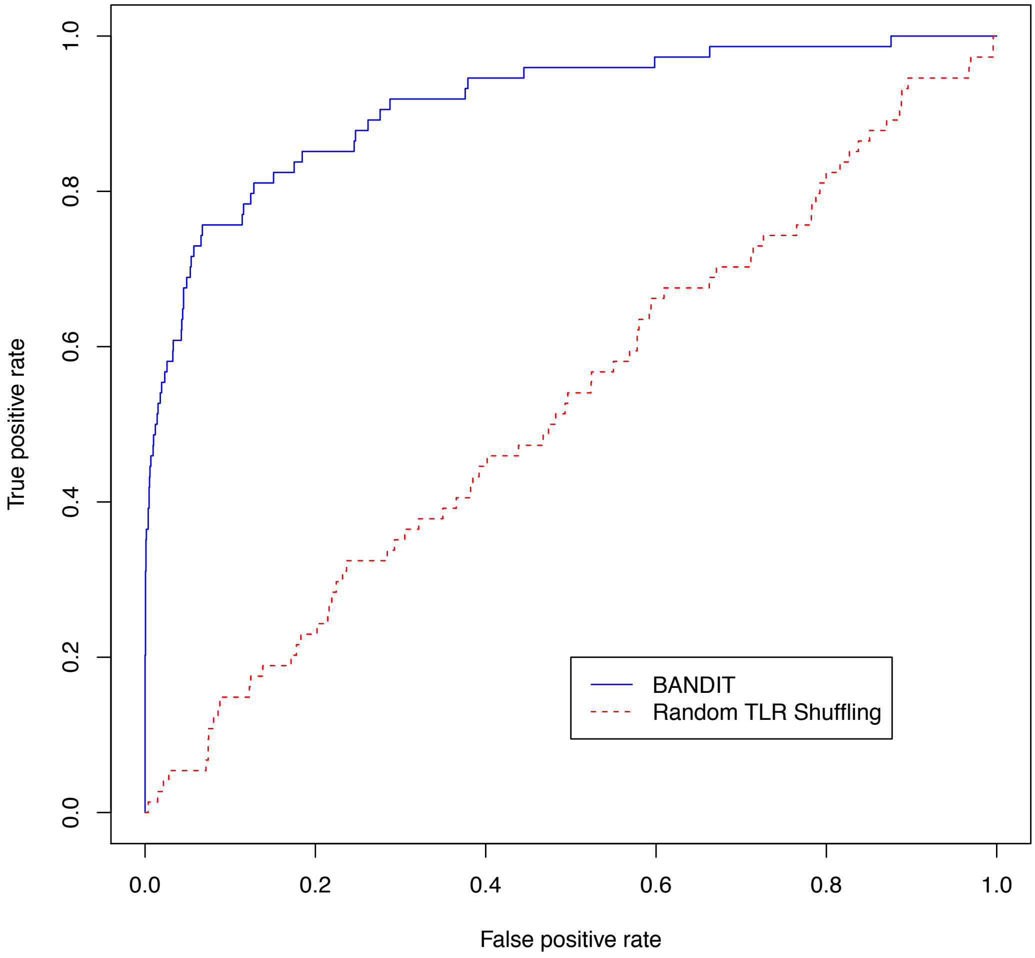

Supplementary Figure 3: BANDIT's AUROC Outperforms Random Shuffling
AUROCs for BANDIT compared to a random shuffling of total likelihood ratios (TLRs)

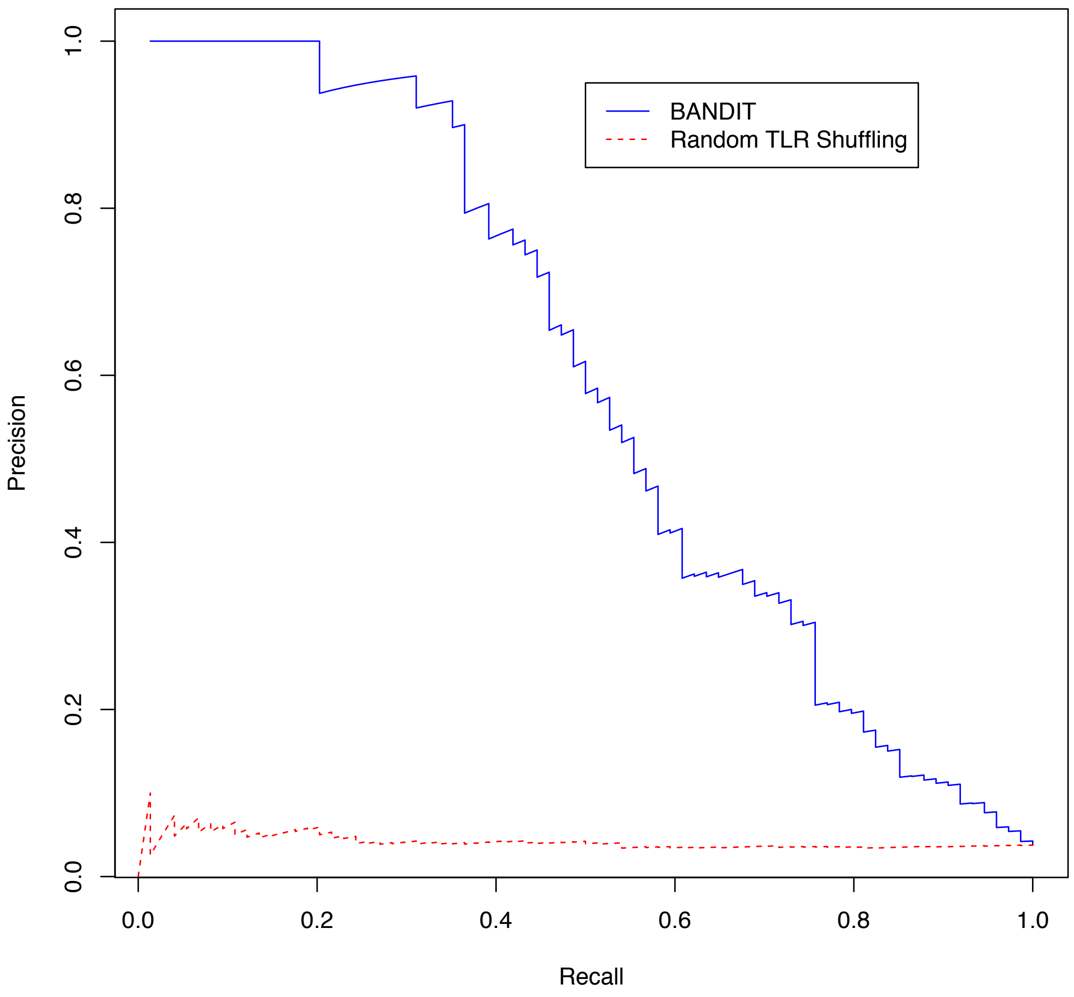

Supplementary Figure 4: BANDIT's AUPRC Outperforms Random Shuffling
AUPRCs for BANDIT compared to a random shuffling of total likelihood ratios (TLRs)

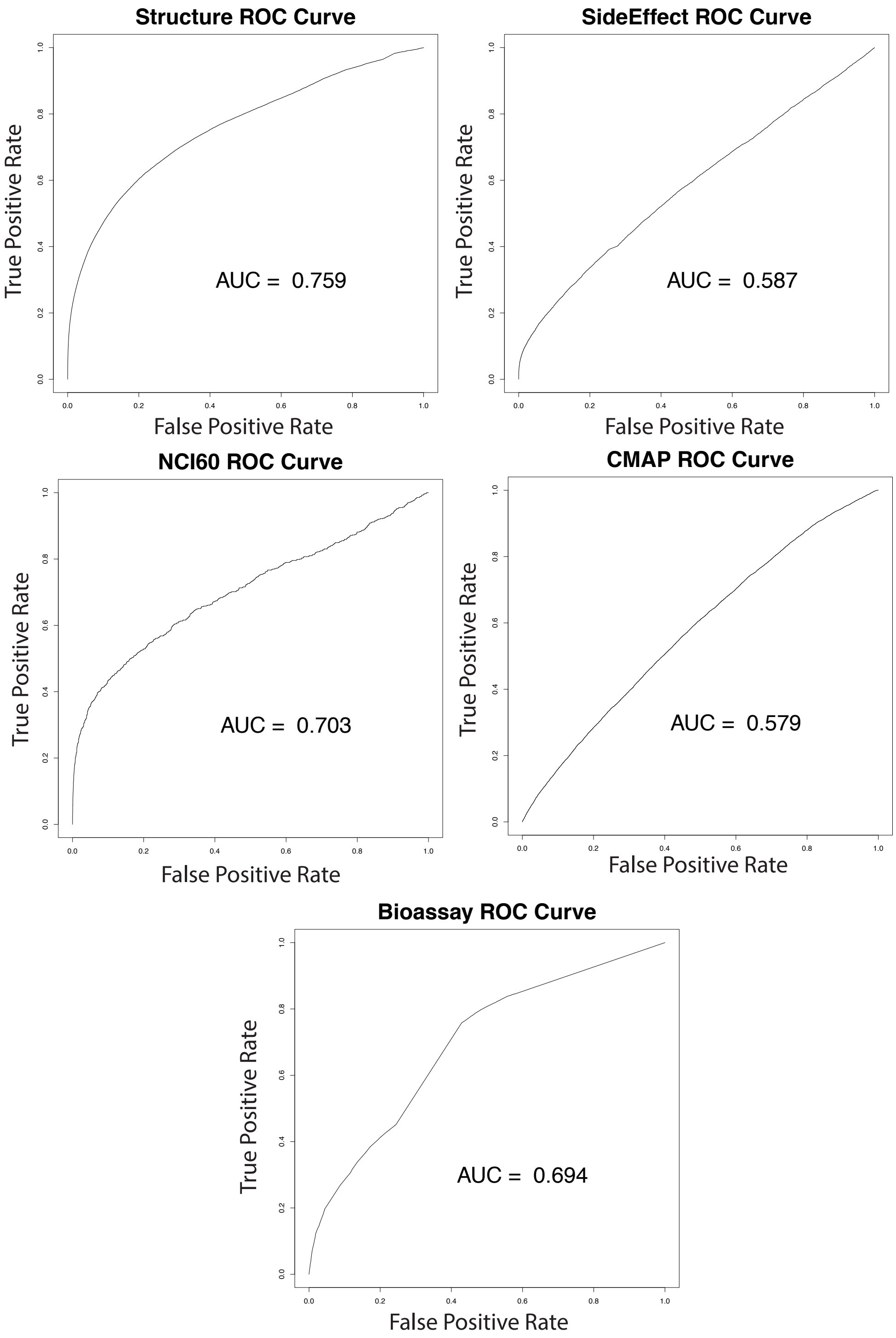

Supplementary Figure 5: Predictive power of individual data types

Area under the receiver-operating curve for different data type specific likelihood ratios.

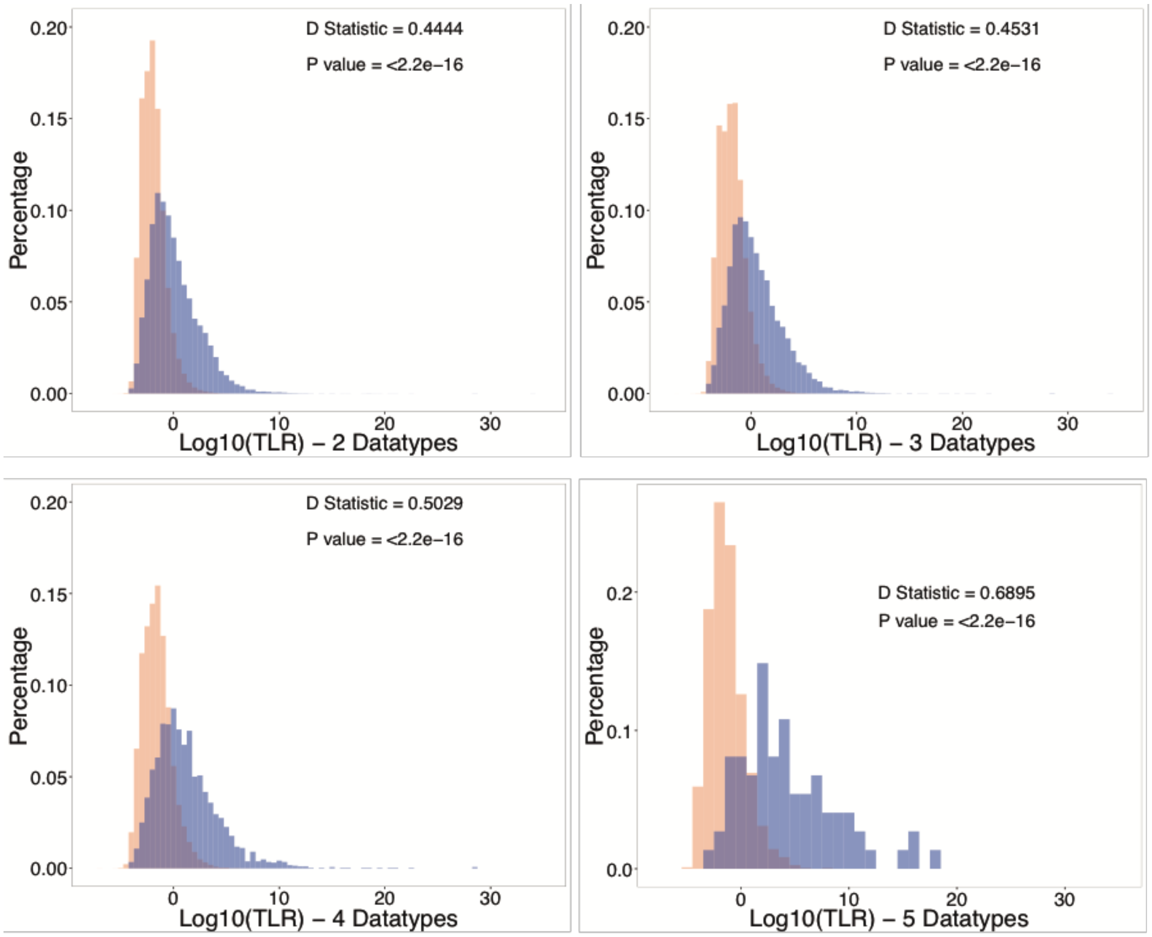

Supplementary Figure 6: BANDIT's TLR output accurately separates drug pairs with shared targets

BANDIT's TLR output accurately separates drug pairs with shared targets– Distributions of TLR scores across two sets – drug pairs known to share a target and those with no known shared targets – with increasing requirements on the number of overlapping data types. P values and D statistics were calculated using the Kolmogorov-Smirnov test. Blue = Shared target drug pairs; Pink = No shared target pairs.

.

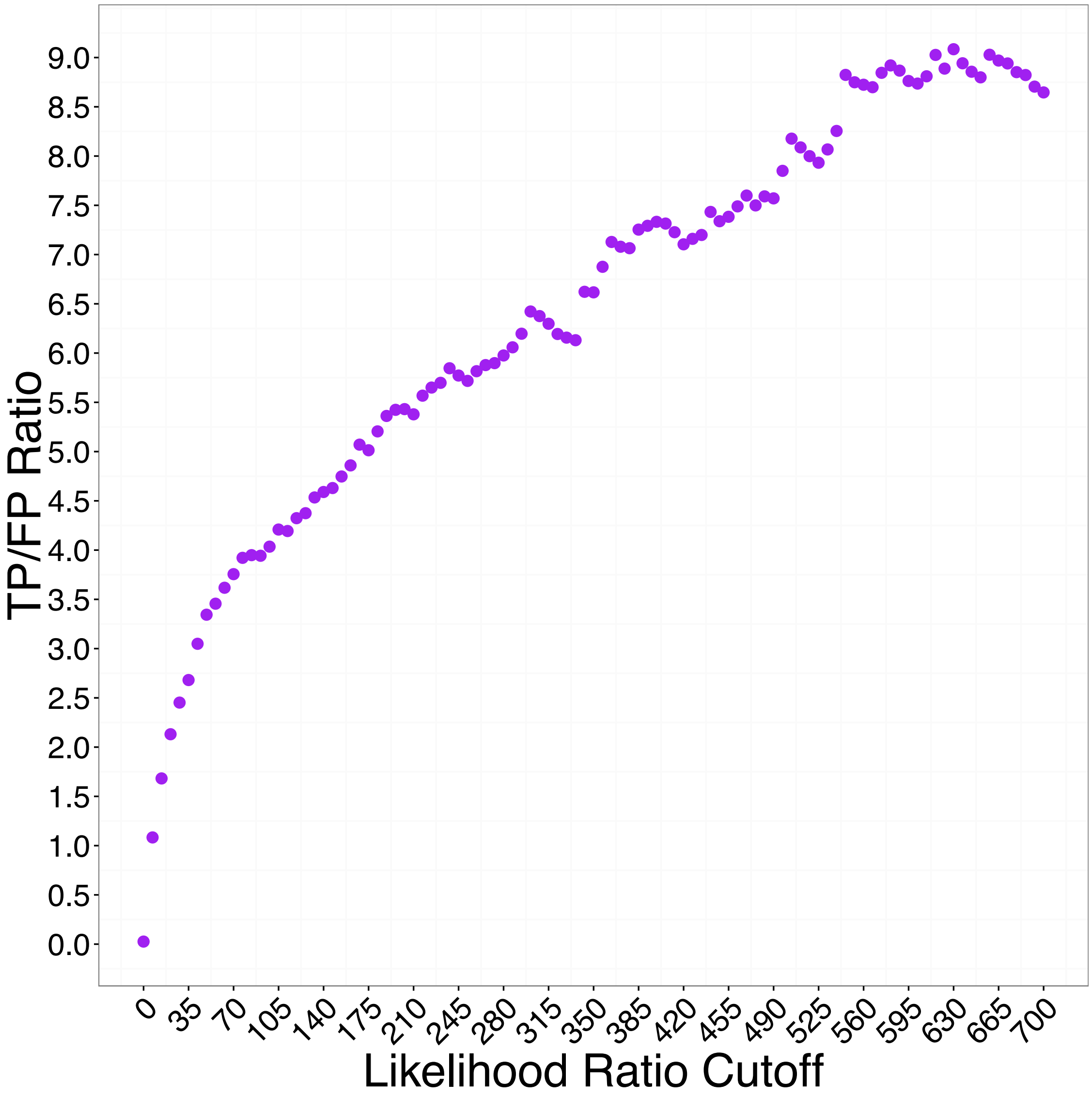

Supplementary Figure 7: TP/FP ratio increases with TLR cutoff to max value of 9
Ratio of true positives to false positives at different likelihood ratio cutoffs with a plateau at TP/FP = 9 at TLR > 550.

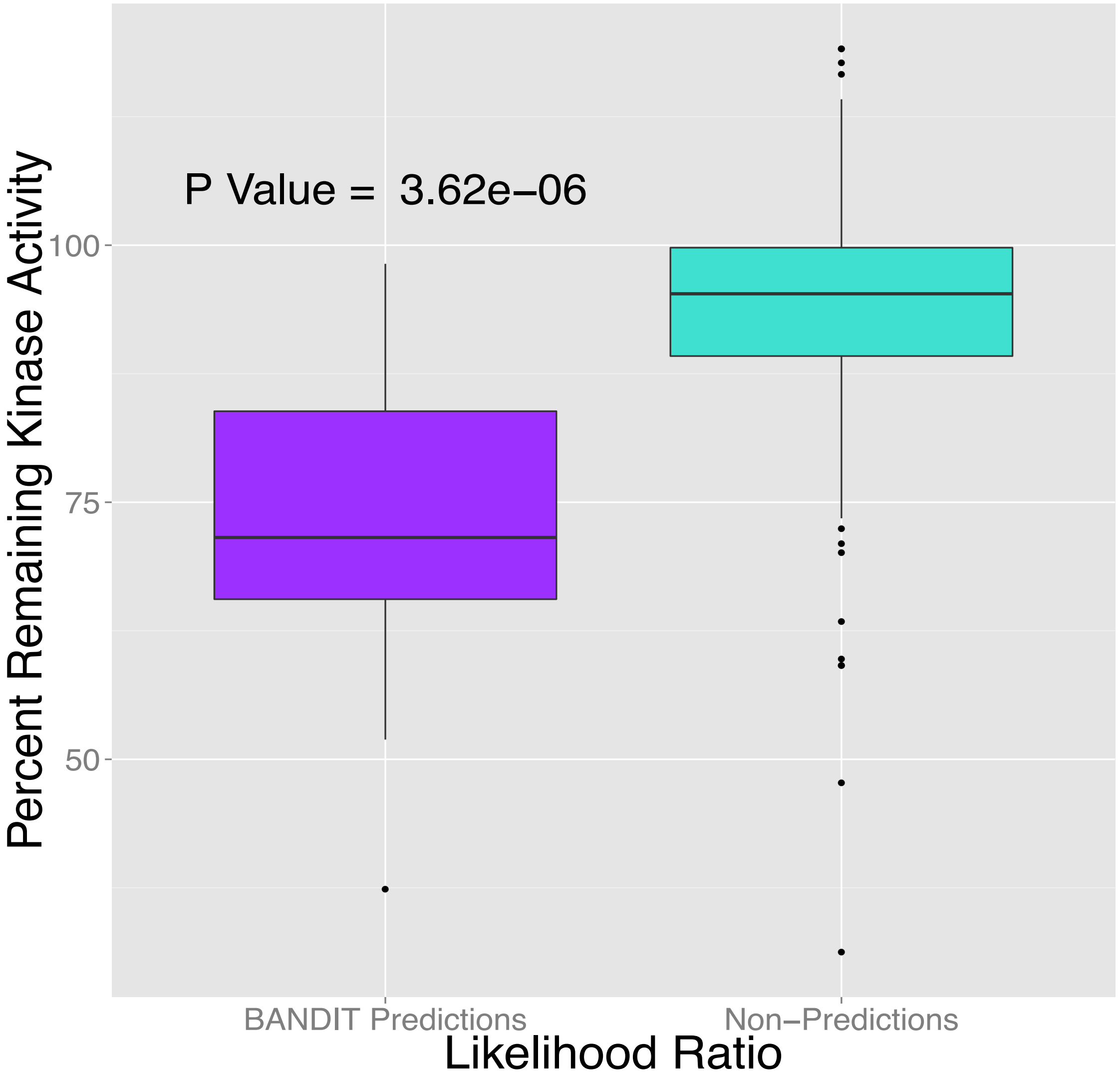

Supplementary Figure 8: BANDIT can replicate results from an experimental kinase screen

Boxplot showing the distributions of "% inhibition" across BANDIT predictions and non-predictions. P value was calculated using a Mann Whitney test.

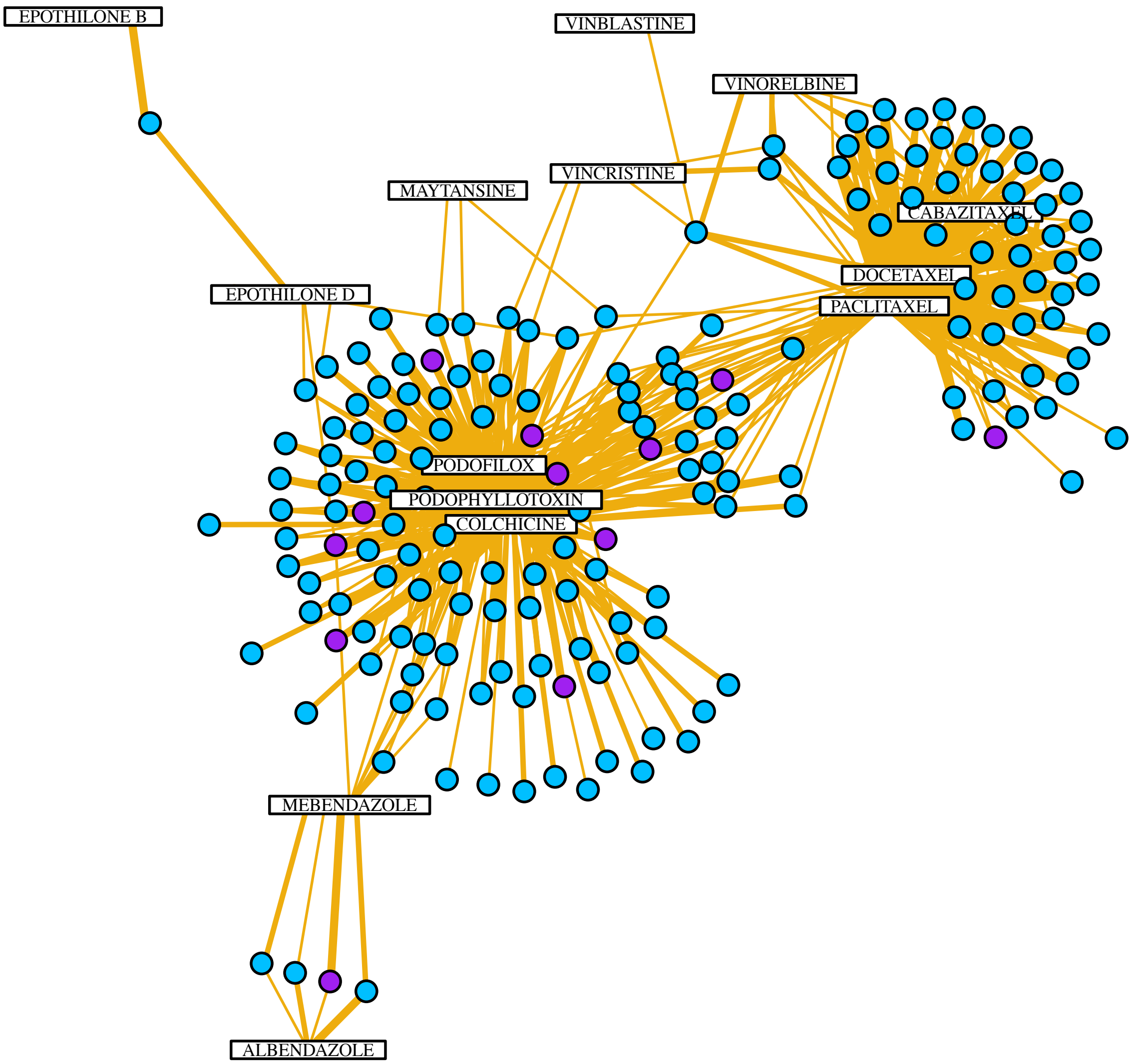

Supplementary Figure 9: Known microtubule inhibitors cluster based on mechanism of action

Network of known microtubules inhibitors and orphan molecules predicted to target molecules. Named boxes represent known inhibitors, blue circles represent predicted inhibitors, and purple circles represent predicted inhibitors that were validated experimentally.

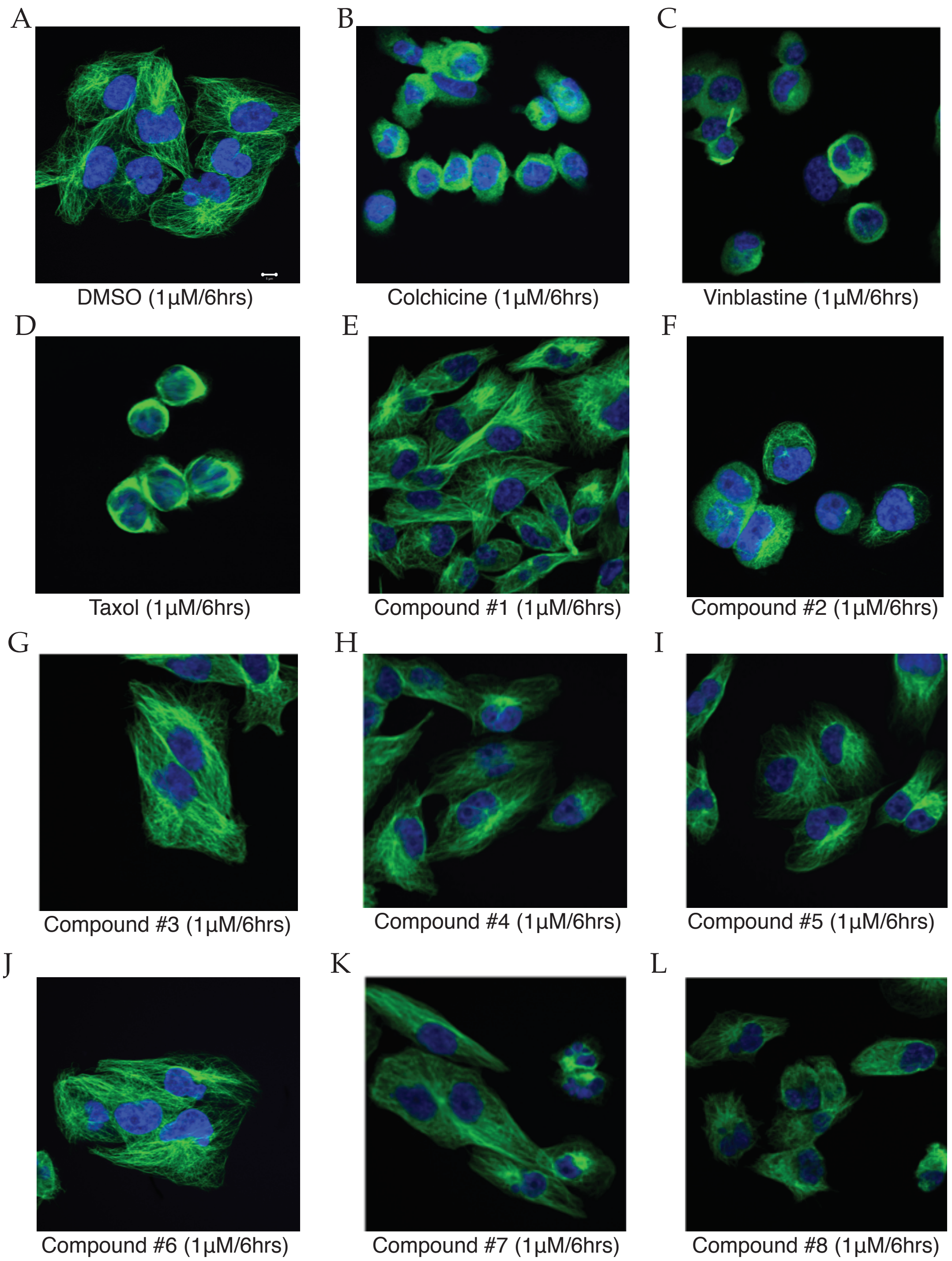

Supplementary Figure 10: BANDIT predicted microtubule inhibitors validated using in-vitro imaging

Effect of various compounds (1µM) on microtubule integrity of MDA-MB-231 cells after 6 hours of treatment: A) DMSO (Scale bar: 5 µm), B) Colchicine, C) Vinblastine, D) Taxol, and E-L) Compounds #1-8

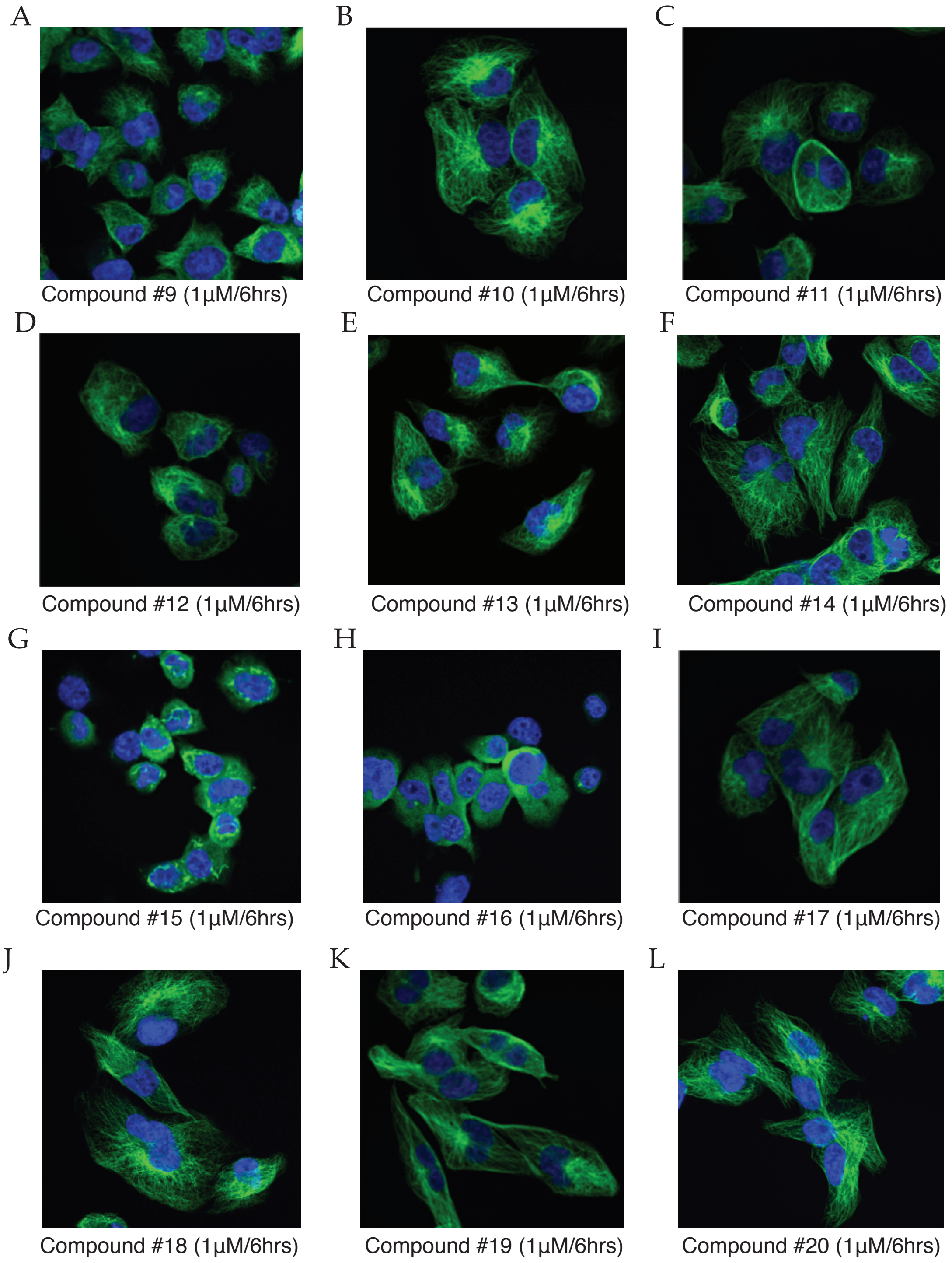

Supplementary Figure 11: BANDIT predicted microtubule inhibitors validated using in-vitro imaging

Effect of various compounds (1µM) on microtubule integrity of MDA-MB-231 cells after 6 hours of treatment: A-L) Compounds #9-20

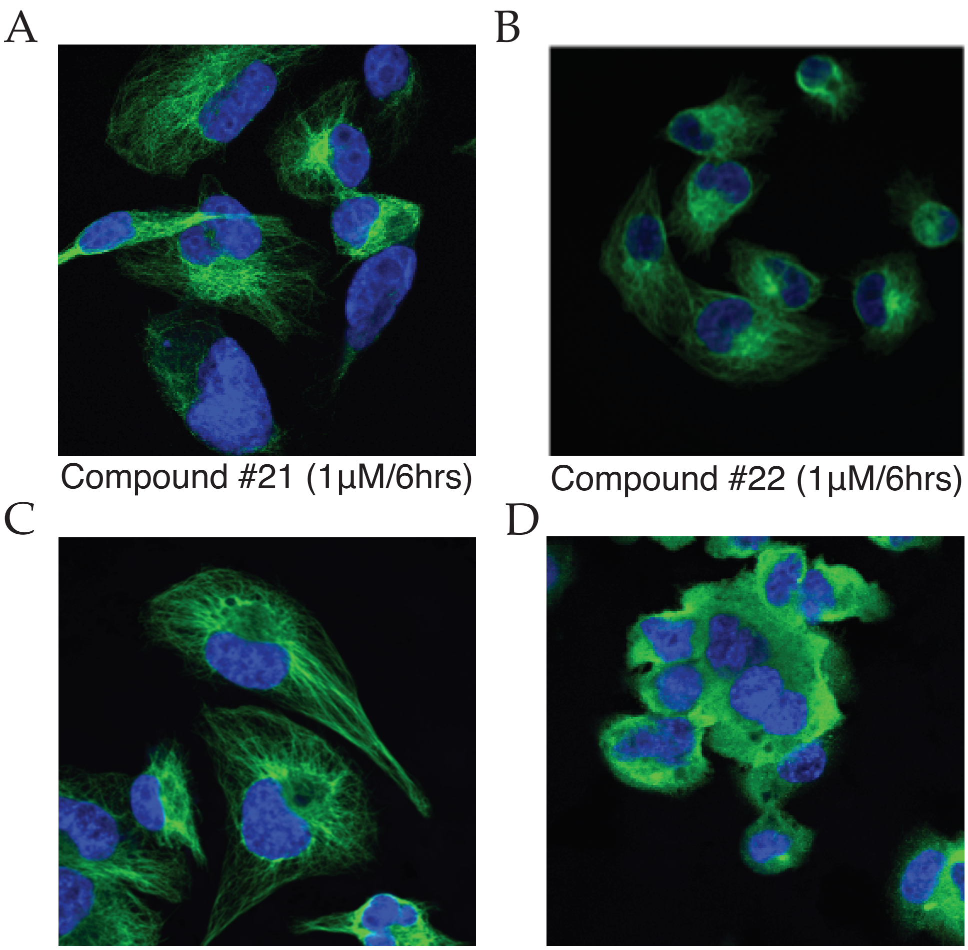

Compound #21 (1µM/6hrs)
Compound #22 (1µM/6hrs)
Compound #23 (1µM/6hrs)
Compound #24 (1µM/6hrs)

Supplementary Figure 12: BANDIT predicted microtubule inhibitors validated using in-vitro imaging

Effect of various compounds (1µM) on microtubule integrity of MDA-MB-231 cells after 6 hours of treatment: A-D) Compounds #21-24

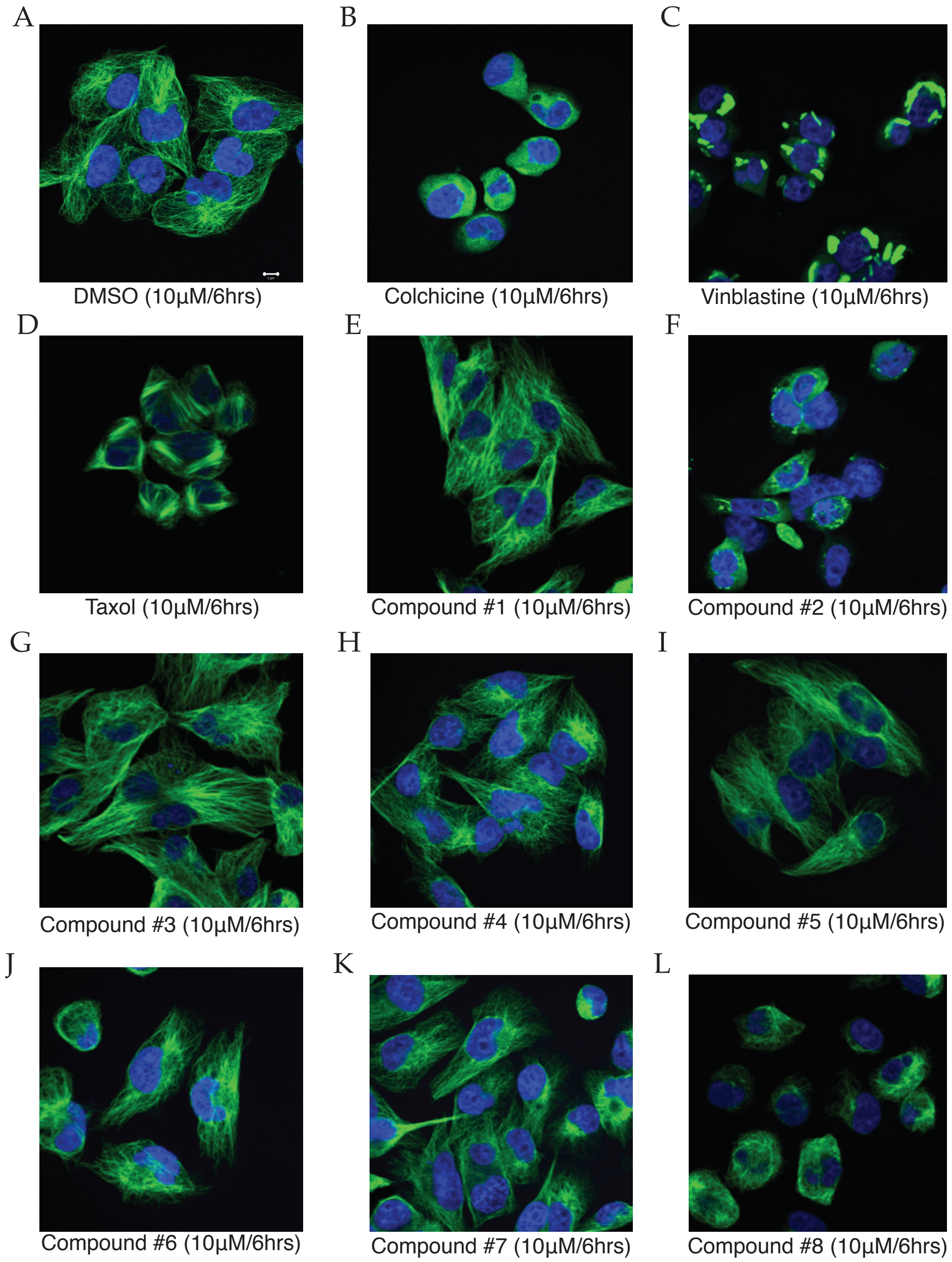

Supplementary Figure 13: BANDIT predicted microtubule inhibitors validated using in-vitro imaging

Effect of various compounds (10µM) on microtubule integrity of MDA-MB-231 cells after 6 hours of treatment: A) DMSO (Scale bar: 5 µm), B) Colchicine, C) Vinblastine, D) Taxol, and E-L) Compounds #1-8

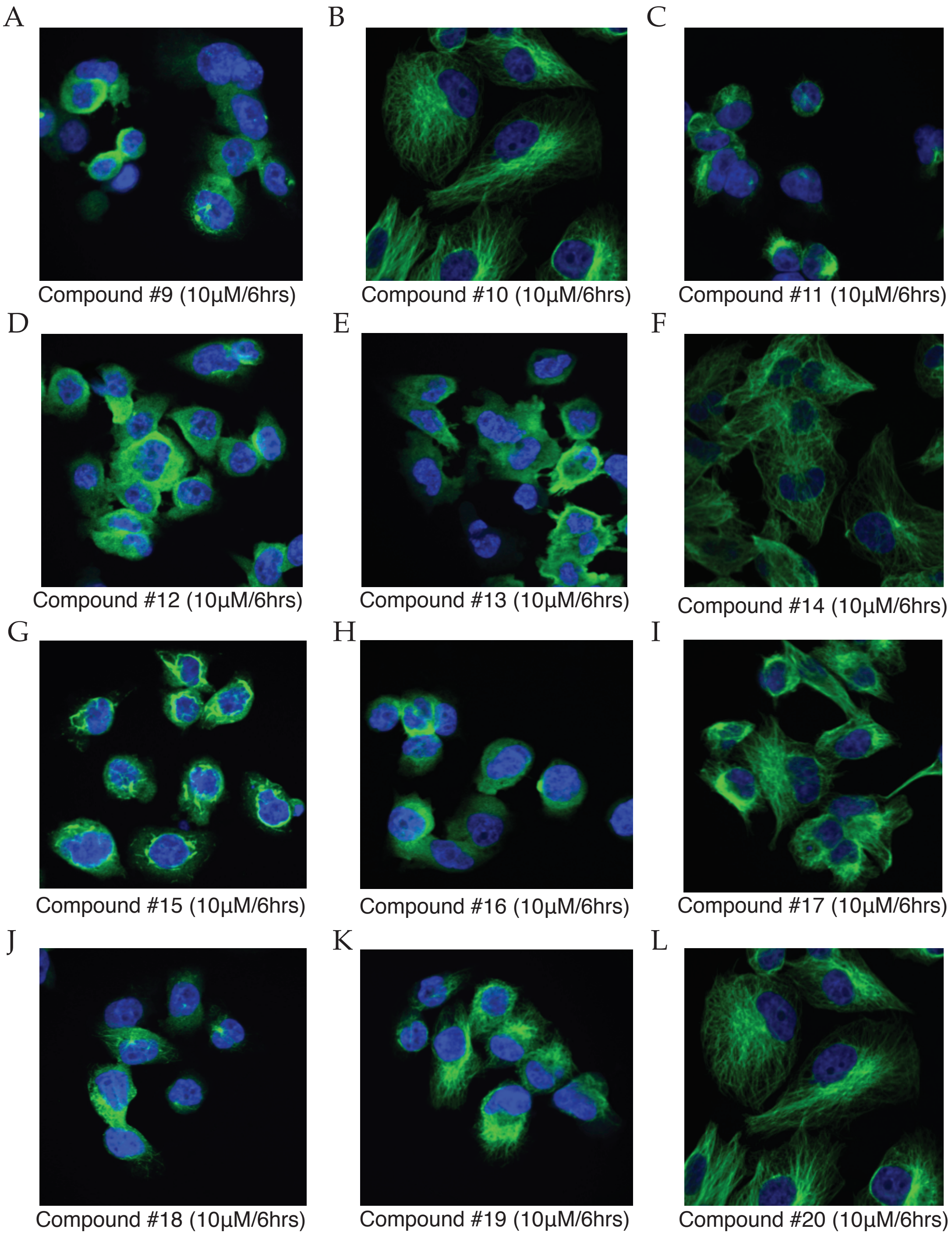

Supplementary Figure 14: BANDIT predicted microtubule inhibitors validated using in-vitro imaging

Effect of various compounds (10µM) on microtubule integrity of MDA-MB-231 cells after 6 hours of treatment: A-L) Compounds #9-20

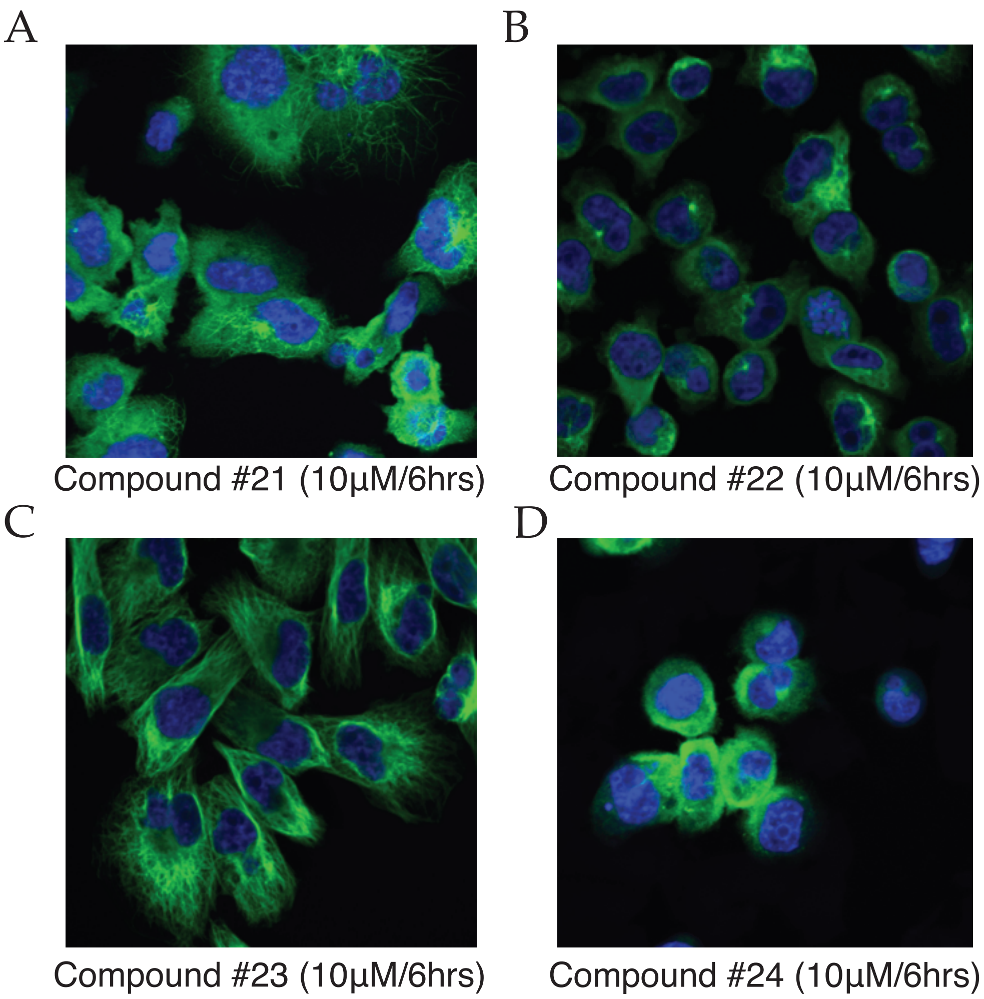

Supplementary Figure 15: BANDIT predicted microtubule inhibitors validated using in-vitro imaging

Effect of various compounds (10µM) on microtubule integrity of MDA-MB-231 cells after 6 hours of treatment: A-D) Compounds #21-24

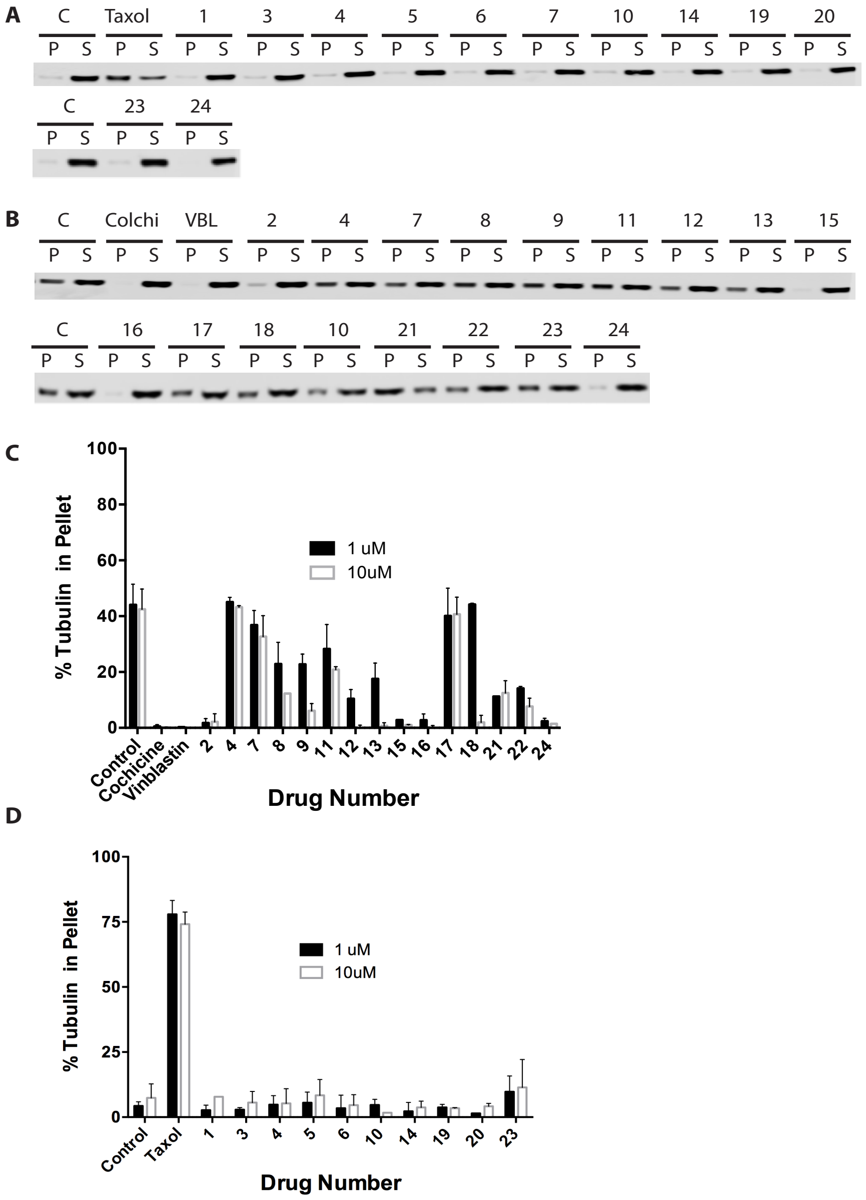

Supplementary Figure 16: Quantification experiments backed up the activity of BANDIT predicted inhibitors on microtubules

Effect of drugs microtubule integrity of MDA-MB-231 cells after 6 hours of treatment. A) Western blots for supernatant (S) and sellet (P) fractions were examined by SDS-PAGE for MDA-MB-231 cells after 6 hours (1μM) of treatment for polymerizing drugs, B) Western blots for supernatant (S) and sellet (P) fractions were examined by SDS-PAGE for MDA-MB-231 cells after 6 hours (1μM) of treatment for depolymerizing drugs, C) Bar graph showing the % of tubulin in the pellet compared to the supernatant (averaged over three independent replicates) for depolymerizing drugs at 1 and 10 μM, and D) Bar graph showing the % of tubulin in the pellet compared to the supernatant (averaged over three independent replicates) for polymerizing drugs at 1 and 10 μM.

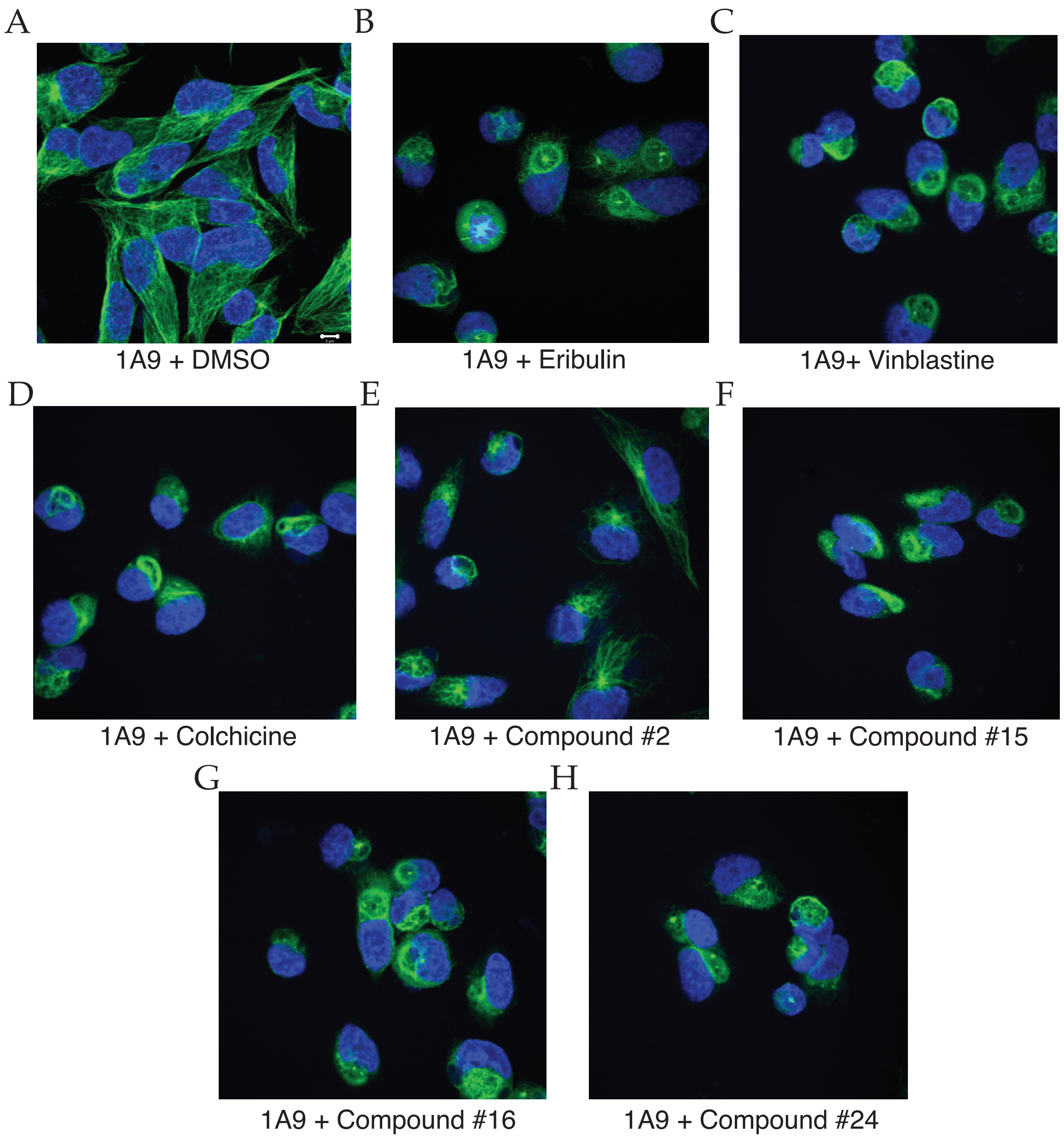

Supplementary Figure 17: Effect of various compounds on the microtubule integrity of 1A9 cells after 6 hours of treatment

A) Control with DMSO (Scale bar: 5 µm), 100nM of B) Eribulin, C) Vinblastine, D) and Colchicine, and 1µM of E) Compound #2, F) Compound #15, G) Compound #16 and H) Compound #24.

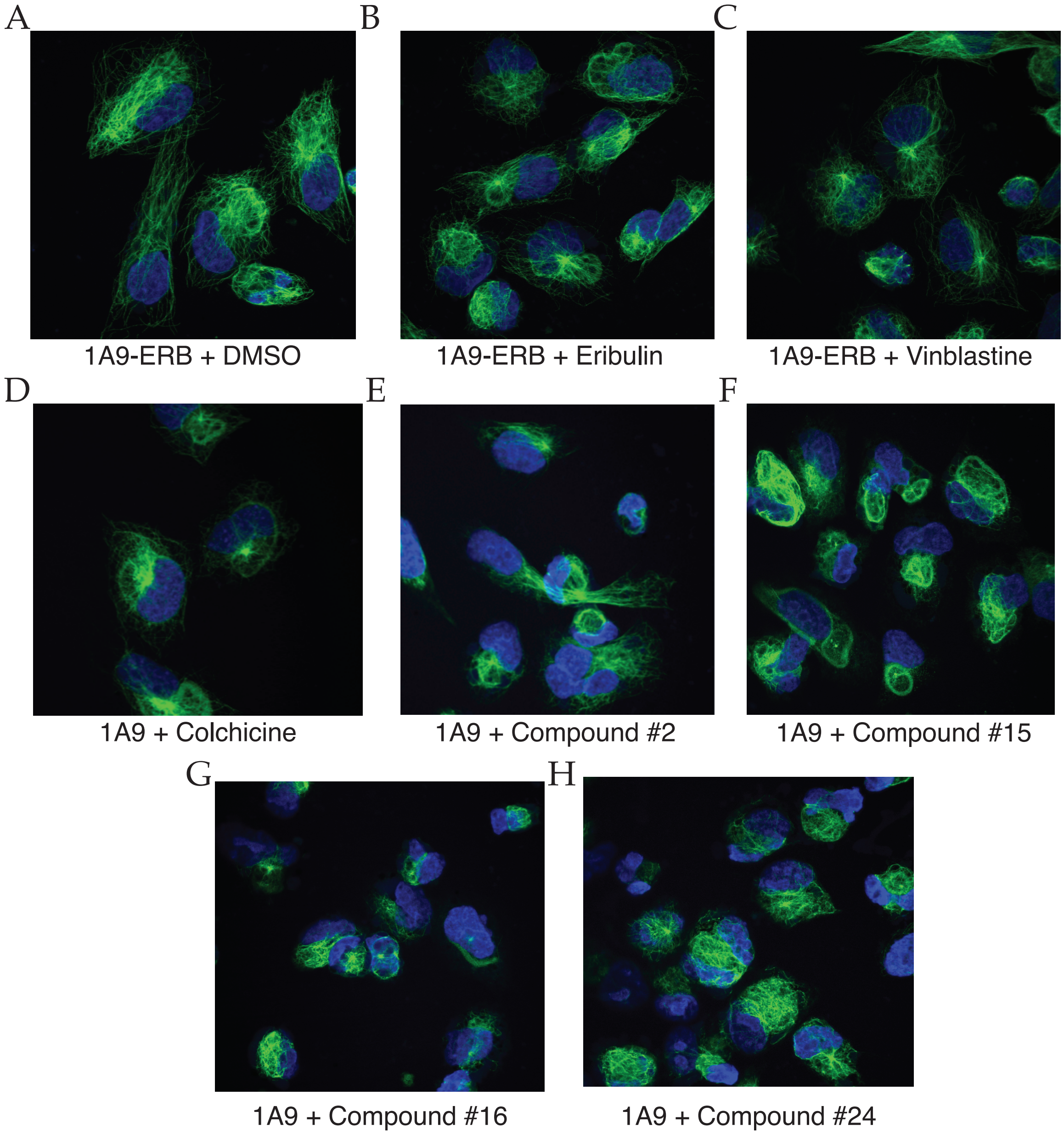

Supplementary Figure 18: A set of the validated microtubule inhibitors can act on resistant cell lines

Effect of various compounds on the microtubule integrity of 1A9-ERB cells after 6 hours of treatment: A) Control with DMSO, 100nM of B) Eribulin, C) Vinblastine, D) and Colchicine, and 1µM of E) Compound #2, F) Compound #15, G) Compound #16 and H) Compound #24.

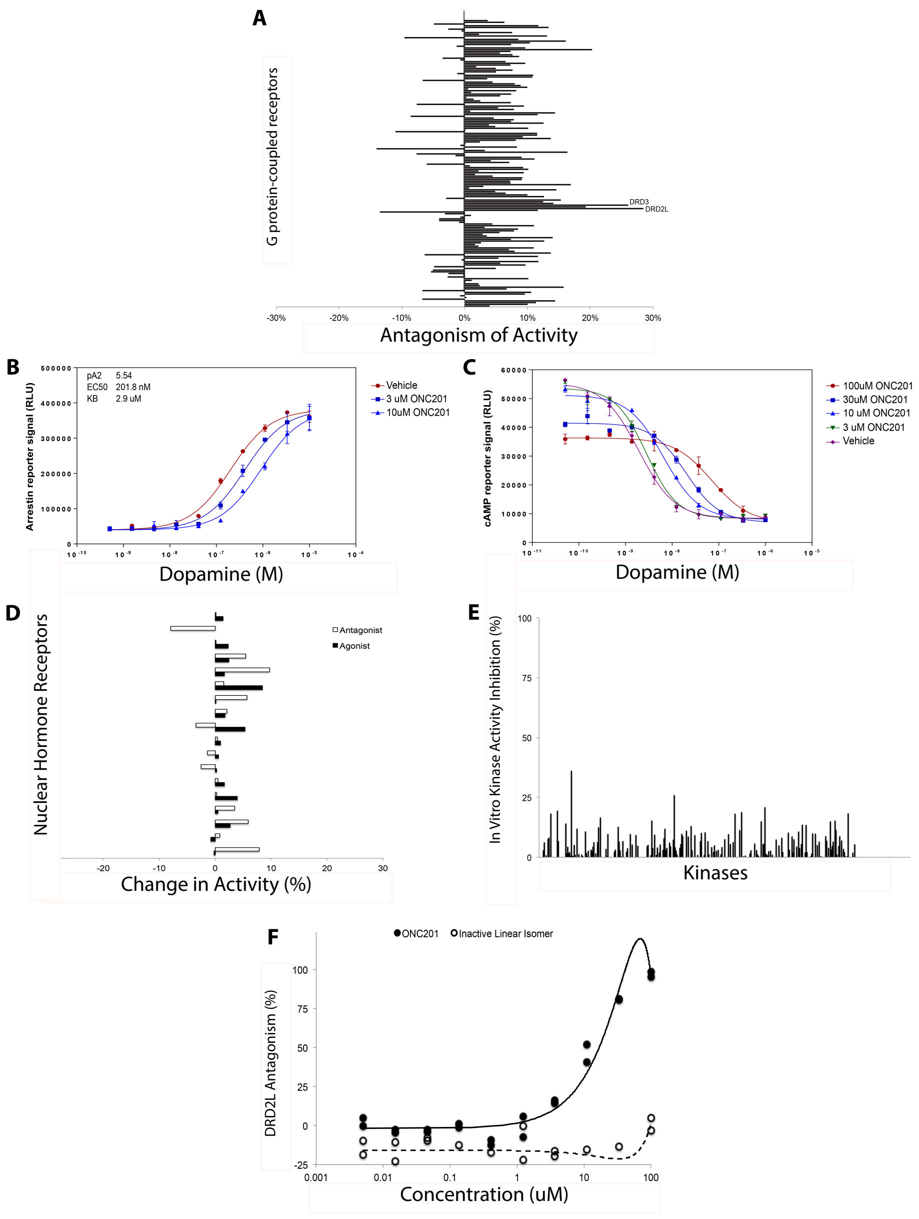

Supplementary Figure 19: The antagonism of DRD2 by ONC201 is highly specific

A) Antagonism of GPCRs using an arrestin recruitment reporter assay (10μM). B) Competition of ONC201-mediated antagonism of DRD2L by dopamine in arrestin recruitment or (C) cAMP modulation reporters. D) Antagonism or agonism of nuclear hormone receptors by ONC201 (2 or 20uM) using a nuclear translocation reporter assay. (E) Inhibition of in vitro kinase enzymatic activity by ONC201 (1 uM). F) DRD2L antagonistic activity of ONC201 or a linear constitutional isomer of ONC201 that has no biological activity using an arrestin recruitment reporter assay.

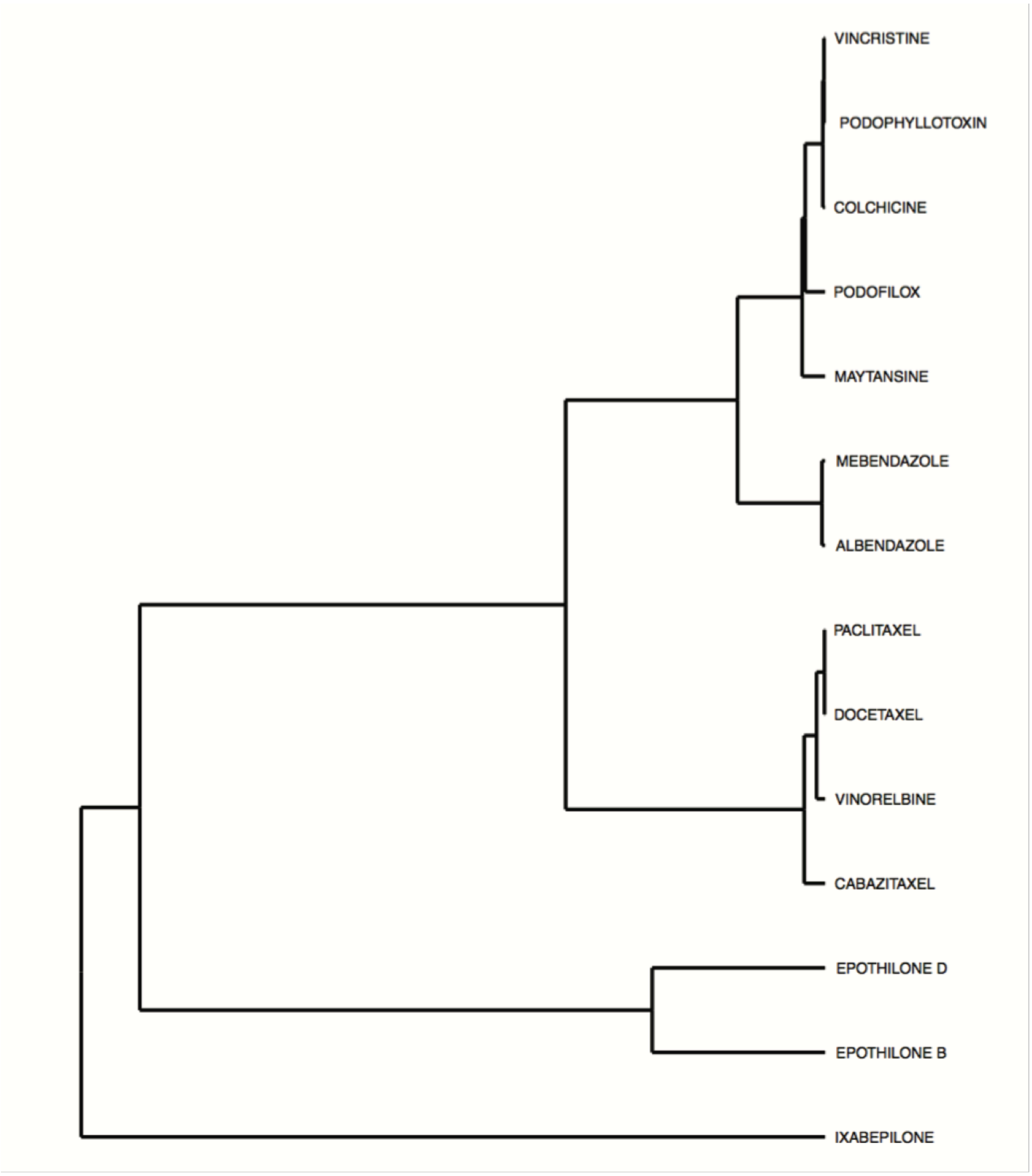

Supplementary Figure 20: Mechanism of action based clusters persist even when structural information is removed

Hierarchical cluster of known microtubule inhibitors based on BANDIT output minus structural features

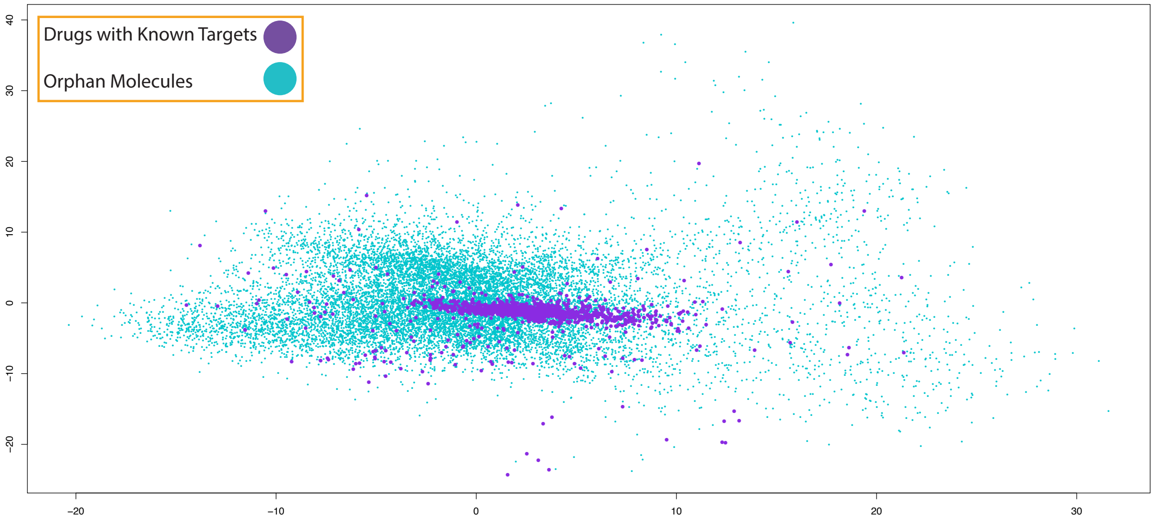

Supplementary Figure 21: Multi-dimensional scaling of both known and orphan small molecules based on BANDIT outputs.

SUPPLEMENTARY TABLES

Number of Data Types Included	Total Combinations of Different Data Types Included	Average AUROC	Standard Deviation in AUROC
5	1	.885	NA
4	5	.825	.025
3	10	.788	.035
2	10	.737	.054
1	5	.664	.079

Supplementary Table 1: Average AUROC and standard deviation based on number of included data types in BANDIT.

Compound #	NSC #	Validated Anti-microtubule Activity (by pellet assay or microscopy)
1	15628	
2	33410	Yes
3	82591	Yes
4	116555	
5	123127	
6	129185	
7	141540	
8	211489	Yes
9	238159	Yes
10	246131	
11	313680	Yes
12	328403	Yes
13	335989	Yes
14	342443	
15	366078	Yes
16	406042	Yes
17	648543	
18	665485	Yes
19	665488	Yes
20	667931	
21	667932	Yes
22	673622	Yes
23	251677	
24	90636	Yes

Supplementary Table 2: List of BANDIT predicted microtubule inhibitors that we were able to obtain from the NCI DTP.

Drug	1A9 (nM)	1A9-ERB (nM)	Fold Resistance[a]
Eribulin	0.34 ± .02	2397 ± 92	7050
Vinblastine	0.08 ± .01	208 ± 23	2600
Colchicine	11 ± .4	560 ± 13	51
Drug No. 15	20 ±5.4	86 ± 14.8	4.3

a=Ratio of GI50 values of resistant/parental cells (1A9-ERB/1A9)

Supplementary Table 3: Newly identified compound can circumvent resistance in cytotoxic assays

Cytotoxic activity of various drugs tested in a 72-hr anti-proliferative assay against parental 1A9 and eribulin resistant (1A9-ERB) cells. IC50 (nM) values for each drug indicate the concentration that kills 50% of the cells in 72 hr

Method	Method Type	Integrated Data Types	Test Set Used	Accuracy /AUC	Predicted DRD2 for ONC201
BANDIT	Bayesian machine learning	Side Effects, Chemical Structure, Transcriptional Response, Bioassays, Growth Inhibition Screening	DrugBank	• 0.89 AUC<br>• 76% accuracy for top predictions (top .166% of scores)	Yes
Campillos et al	Similarity network	Side Effects	DrugBank, Matador, & PDSP	• 47% accuracy for top predictions (top .166% of scores)	NA (Unable to test as ONC201 is still in development and does not have well profiled side effects)
Wang et al	Batch corrected GSEA	Transcriptional Response	DrugBank	0.66 AUC	No
Caniza et al – SIMui	Graph based GO similarity	MESH Ontology Terms	DrugBank	0.69 AUC	NA (Unable to test as ONC201 is still in development and does not have well profiled MESH Ontology terms)
Caniza et al – Resnik	Semantic similarity	MESH Ontology Terms	DrugBank	0.67 AUC	NA (Unable to test as ONC201 is still in development and does not have well profiled MESH Ontology terms)
Caniza et al – SLMgic	Graph based GO similarity	MESH Ontology Terms	DrugBank	0.65 AUC	NA (Unable to test as ONC201 is still in development and does not have well profiled MESH Ontology terms)
Caniza et al – Lin	Semantic similarity	MESH Ontology Terms	DrugBank	0.55 AUC	NA Unable to test as ONC201 is still in development and does not have well profiled MESH Ontology terms)
Caniza et al – Jiang and Conrath	Semantic similarity	MESH Ontology Terms	DrugBank	0.52 AUC	NA (Unable to test as ONC201 is still in development and does not have well profiled MESH Ontology terms)
Keiser et al	Ligand based similarities	Chemical Structure	MDDR, WOMBAT	NA	No
Nickel et al	Similarities based on structure and ATC codes	Chemical Structure, ATC Code	SuperTarget, ChEMBL, BindingDB	NA	No

Supplementary Table 4: Comparison of BANDIT's accuracy to other published methods
